# Brain Ischemia as a Prelude to Alzheimer's Disease

**DOI:** 10.3389/fnagi.2021.636653

**Published:** 2021-02-18

**Authors:** Ryszard Pluta, Sławomir Januszewski, Stanisław J. Czuczwar

**Affiliations:** ^1^Laboratory of Ischemic and Neurodegenerative Brain Research, Mossakowski Medical Research Institute, Polish Academy of Sciences, Warsaw, Poland; ^2^Department of Pathophysiology, Medical University of Lublin, Lublin, Poland

**Keywords:** brain ischemia, neurodegeneration, amyloid protein precursor, secretases, presenilins, tau protein, folding proteins, Alzheimer's disease

## Abstract

Transient ischemic brain injury causes massive neuronal death in the hippocampus of both humans and animals. This was accompanied by progressive atrophy of the hippocampus, brain cortex, and white matter lesions. Furthermore, it has been noted that neurodegenerative processes after an episode of ischemia-reperfusion in the brain can continue well-beyond the acute stage. Rarefaction of white matter was significantly increased in animals at 2 years following ischemia. Some rats that survived 2 years after ischemia developed severe brain atrophy with dementia. The profile of post-ischemic brain neurodegeneration shares a commonality with neurodegeneration in Alzheimer's disease. Furthermore, post-ischemic brain injury is associated with the deposition of folding proteins, such as amyloid and tau protein, in the intracellular and extracellular space. Recent studies on post-ischemic brain neurodegeneration have revealed the dysregulation of Alzheimer's disease-associated genes such as amyloid protein precursor, α-secretase, β-secretase, presenilin 1, presenilin 2, and tau protein. The latest data demonstrate that Alzheimer's disease-related proteins and their genes play a key role in the development of post-ischemic brain neurodegeneration with full-blown dementia in disease types such as Alzheimer's. Ongoing interest in the study of brain ischemia has provided evidence showing that ischemia may be involved in the development of the genotype and phenotype of Alzheimer's disease, suggesting that brain ischemia can be considered as a useful model for understanding the mechanisms responsible for the initiation of Alzheimer's disease.

## Introduction

Presently, brain ischemia and Alzheimer's disease create a huge burden to the healthcare system and caregivers due to the lack of causal treatment. Both the diseases are the main causes of irreversible disability and dementia worldwide (Ballard et al., 2011; Pluta et al., 2011, 2020b,c,d; Kim and Lee, 2018; Lo et al., 2019), and there is a risk of stroke or Alzheimer's disease is one in three persons (Seshadri and Wolf, 2007). With increasing numbers of aging in the world, the number of subjects with dementia is forecast to reach 82 million by 2030 and 152 million by 2050 (Ballard et al., 2011). There are no causal treatments that could stop the development of dementia in both patients after stroke and Alzheimer's disease. For this reason, there is a lot of pressure to increase the understanding of the mechanisms of post-ischemic brain neurodegeneration in connection with its recommended relationship with Alzheimer's disease (Pluta, 2007b, 2019; Pluta et al., 2011, 2013a,b; Salminen et al., 2017) and to make causal therapy available (Ułamek-Kozioł et al., 2020a). It is noteworthy that more and more new clinical and experimental studies indicate epidemiological and neuropathological links connecting ischemic brain neurodegeneration with the genotype and phenotype of Alzheimer's disease. Human investigations have revealed that Alzheimer's disease is a risk factor for stroke (Chi et al., 2013; Tolppanen et al., 2013) and *vice versa* (Gamaldo et al., 2006), indicating that the same or closely related pathological mechanisms may be involved in the development of both disorders. Animal studies have also presented a synergistic link between brain ischemia and Alzheimer's disease, leading to an increased risk of cognitive decline and development of Alzheimer's disease-type dementia (De la Tremblaye and Plamondon, 2011; Kiryk et al., 2011; Li et al., 2011; Cohan et al., 2015; Traylor et al., 2016; Salminen et al., 2017). The main cause of ischemic stroke in humans is atherosclerosis (Roher et al., 2004; Beach et al., 2007). Atherosclerosis is also associated with Alzheimer's disease (Farkas and Luiten, 2001; Thal et al., 2003; Beach et al., 2007). At least 33% cases of Alzheimer's disease have neuropathological changes resulting from small vessel arteriosclerosis (Kalaria, 2002). Atherosclerosis has been found to coexist with cerebral amyloid angiopathy and it also correlates well with cognitive decline (Thal et al., 2003; Pluta et al., 2009). On the other hand, the increased level of amyloid in the post-ischemic brain causes the accumulation of amyloid not only in the brain tissue, but also in the vessel wall, causing the development of cerebral amyloid angiopathy (Pluta et al., 2009; Hecht et al., 2018). Reduction in the length of cerebral vessels post-ischemia or impaired cerebral blood flow in the brain as a result of vasoconstriction (Wisniewski et al., 1995) and/or the development of cerebral amyloid angiopathy (Pluta et al., 2009; Hecht et al., 2018) not only limits the transport of energy substrates and the supply of oxygen and nutrients to the brain through the blood–brain barrier after ischemia, but also reduces the clearance of potential neurotoxins from the brain, such as amyloid (Hecht et al., 2018). This leads to the idea that brain vascular diseases, such as ischemic brain episode, may make the regions in the brain more susceptible to Alzheimer's disease pathology, due to impaired clearance of amyloid from the brain (Farkas and Luiten, 2001) and dysfunctional tau protein. Alternatively, post-ischemic brain neurodegeneration and Alzheimer's disease may finally represent independent but convergent common pathological mechanisms, and can therefore be expected to have common proteomic and genomic risk factors (Traylor et al., 2016; Pluta et al., 2019a,b; Ułamek-Kozioł et al., 2020b).

## Neuropathology in Post-Ischemic Neurodegeneration

The death of neurons in the CA1 area of hippocampus develops during 7 days following experimental ischemic episode (Pluta, 2002a). Extended survival, following ischemic brain injury up to 2 years, triggers additional pathology in neuronal cells in the hippocampal CA3 region which is resistant to ischemic injury (Pluta et al., 2009). The same changes are observed in post-stroke cases (Gemmell et al., 2012, 2014). In cortex, layers 3, 5, and 6 presented massive neuronal changes (Pluta, 2000a, 2002a,b), but changes in striatum were related to medium-sized neurons. Two years after ischemia, in addition to localized neuron loss, a variety of pathological stages of neuronal cells were observed (Pluta et al., 2009). The neuronal loss took the form of chronic neuronal damage. The other alterations with acute character were noted in those areas of the brain, which were not involved in primary ischemic pathology, i.e. in CA2, CA3, and CA4 areas of the hippocampus (Pluta et al., 2009).

Brain ischemia increases the permeability of the blood–brain barrier to cellular and non-cellular blood elements i.e., platelets and amyloid (Mossakowski et al., 1993, 1994; Pluta et al., 1994a, 2006; Wisniewski et al., 1995; Pluta, 2003, 2005). In post-ischemic leakage of the blood–brain barrier, two facts deserve attention. The first one is very important in terms of amyloid extravasations (Pluta et al., 1996, 1997b) during the development of brain neurodegeneration, and the second is the movement of platelets containing a huge amount of amyloid, which causes toxic and mechanical damage to the brain parenchyma (Pluta et al., 1994c). The ability of the soluble amyloid to pass through the insufficient blood–brain barrier leads to neurotoxic effects on neurons, which may then lead to subsequent increased accumulation of amyloid in post-ischemic brain. Soluble amyloid is delivered to the brain following ischemia from the circulatory system, and as a consequence, it contributes to brain vasoconstriction, amyloidosis, and cerebral amyloid angiopathy, following brain ischemia (Jendroska et al., 1995, 1997; Wisniewski et al., 1995; Pluta et al., 1996, 1999, 2009; Wisniewski and Maslinska, 1996; Lee et al., 2005; Qi et al., 2007; Zetterberg et al., 2011; Liu et al., 2015; Hecht et al., 2018).

Following brain ischemia in regions with massive neuronal alterations, a powerful inflammatory response was observed (Orzyłowska et al., 1999; Pluta, 2000a, 2002a,b; Sekeljic et al., 2012; Radenovic et al., 2020). These data indicate that the increase of inflammatory mediators in microglia and astrocytes is directly related to the selective vulnerability of neurons to ischemia (Orzyłowska et al., 1999; Radenovic et al., 2020). After ischemia, inflammatory factors can trigger a self-sustaining cycle that leads the post-ischemic brain to neurodegeneration. Interleukin-1 is a key player that stimulates ischemic neurons to amyloidogenic processing of the amyloid protein precursor along with the induction of inflammatory factors. These processes induce changes in neurons and their loss, with irreversible interruption of the neuronal network. Consequently, this neuropathology activates microglial cells, which leads to self-propagation of the inflammatory cycle. Furthermore, it is evident that the amyloid, which is generated following ischemia (Pluta et al., 1994b, 2009; Ishimaru et al., 1996), triggers the release of inflammatory mediators by microglia. In the hippocampus, activation of glial cells precedes neuron loss, and it lasts for a long time after an ischemic episode (Sekeljic et al., 2012; Radenovic et al., 2020). Factors released by astrocytes and microglia cells, i.e., matrix metalloproteinases, interleukin-1, and tumor necrosis factor α increase the leakage of the blood–brain barrier (Amantea et al., 2015). Microglia and astrocytes belong to the first line of defense and are activated a few minutes after ischemia (Dabrowska et al., 2019). Increased activation is noted within 2–3 days after ischemia, persisting for years following ischemic brain injury (Sekeljic et al., 2012; Dabrowska et al., 2019; Radenovic et al., 2020).

An increased influx of monocytes in the brain after ischemia was documented within 1 day after ischemia as a result of additional injury to the blood–brain barrier by neuroglial inflammatory factors. An increased number of monocytes in brain tissue were observed up to 7 days following ischemia (Kim et al., 2014). Sooner or later, anti-inflammatory macrophages begin to dominate the brain after ischemia, as they are essential for the processes of regeneration and healing (Gliem et al., 2016). Another type of cells involved in the immune response induced by ischemia are neutrophils which appear in post-ischemic brain parenchyma immediately after the ischemic insult (Price et al., 2004). The neutrophils focus around damaged areas and release inflammatory factors, proteolytic enzymes, and oxygen free radicals that initiate secondary damage to the already damaged brain tissue (Dabrowska et al., 2019; Radenovic et al., 2020). The number of neutrophils appearing in the brain following ischemia directly corresponds to the size of the brain injury (Ahmad et al., 2014; Leinweber et al., 2021). Also, T and B lymphocytes, natural killer cells, and dendritic cells infiltrate the brain after ischemia (Gelderblom et al., 2018; Dabrowska et al., 2019). In addition, mast cells intensify inflammatory responses by releasing tumor necrosis factor α, histamine, heparin, and proteases, i.e., tryptase, chymase, and matrix metalloproteinases, which cause secondary changes in the blood–brain barrier, edema of the brain, and penetration of neutrophils into the ischemic brain tissue (Pluta et al., 1989; Lindsberg et al., 2010; Dabrowska et al., 2019; Leinweber et al., 2021).

Lesions in white matter with the proliferation of neuroglial cells were documented in human and animal brains, after ischemia (Pluta, 2000a, 2002a,b; Fernando et al., 2006; Pluta et al., 2006, 2008, 2009; Scherr et al., 2012; Sekeljic et al., 2012; Thiebaut de Schotten et al., 2014; Zamboni et al., 2017; Radenovic et al., 2020). An episode of cerebral ischemia in animals causes massive damage to the subcortical white matter and corpus callosum (Wakita et al., 1994; Pluta et al., 2006, 2008, 2009). Increased permeability of the blood–brain barrier, after ischemia, allows the movement of inflammatory cells and amyloid from the circulatory system to the brain, which additionally leads to the progression of changes in white matter (Pluta et al., 1996, 1997b, 1999, 2000; Anfuso et al., 2004; Lee et al., 2005; Zetterberg et al., 2011; Liu et al., 2015).

Data suggest that brain ischemia triggers massive neuronal death, especially in brain structures sensitive to ischemia (Pluta, 2000a; Pluta et al., 2009; Bivard et al., 2018). These processes develop both in the early and later stages following ischemia (Pluta, 2002a; Pluta et al., 2009; Bivard et al., 2018; Radenovic et al., 2020). In the years following ischemia, ischemic neurodegenerative processes cause general brain atrophy (Hossmann et al., 1987; Pluta, 2000a; Pluta et al., 2009; Jabłoński et al., 2011; Bivard et al., 2018). Brain autopsy after experimental ischemia, with survival up to 2 years, showed the hallmarks of brain hydrocephalus and dilatation of the subarachnoid space around the brain hemispheres (Hossmann et al., 1987; Pluta, 2000a; Pluta et al., 2009; Jabłoński et al., 2011). Hippocampal atrophy was also observed in humans and animals after ischemia (Pluta, 2000a; Pluta et al., 2009; Gemmell et al., 2012, 2014).

## Amyloid-Related Genes in Post-Ischemic Neurodegeneration

Following experimental brain ischemia, with survival up to 2 years after ischemia, amyloid staining was documented in intracellular and extracellular space (Pluta et al., 1994b, 1997a, 1998, 2012a; Hall et al., 1995; Tomimoto et al., 1995; Ishimaru et al., 1996; Yokota et al., 1996; Pluta, 1997, 2000a,b; Lin et al., 1999, 2001; Sinigaglia-Coimbra et al., 2002; Fujioka et al., 2003; Jabłoński et al., 2011; Pluta and Jabłoński, 2012). Accumulation of amyloid in the extra-cellular space ranged from small dots to diffuse and senile amyloid plaques (Pluta et al., 1994b, 1998, 2000, 2009, 2010; Pluta, 2000a,b; Pluta, 2002a,b; Pluta, 2003, 2005, 2007a; Van Groen et al., 2005). Amyloid plaques were noted in the hippocampus, thalamus, brain cortex, corpus callosum, and around the lateral ventricles. The deposition of amyloid inside neurons and neuroglial cells underscores the possible importance of amyloid in the progress of ischemic brain neurodegeneration (Pluta et al., 1994b; Banati et al., 1995; Palacios et al., 1995; Yokota et al., 1996; Nihashi et al., 2001; Pluta, 2002a,b; Badan et al., 2003, 2004). These observations indicate that following experimental ischemia, amyloid generation may be responsible for additional neurodegenerative mechanisms, which could worsen the outcome after ischemia due to continuous neuronal loss (Pluta et al., 1997a,b; Pluta et al., 1998, 2009, 2011, 2012a,b,c; Jabłoński et al., 2011; Kiryk et al., 2011; Pluta and Jabłoński, 2012). Some data indicate that after ischemic episode, amyloid was generated as a product of neuronal death (Ishimaru et al., 1996). The amyloid is a neurotoxic substance that induces intracellular processes in post-ischemic neurons, astrocytes, and microglia, which further causes extra neurons and neuroglia injury and/or death following ischemia (Giulian et al., 1995; Pluta et al., 2012a).

After ischemia, both diffuse and senile amyloid plaques in the hippocampus and cortex were observed in the human brain (Jendroska et al., 1995, 1997; Wisniewski and Maslinska, 1996; Qi et al., 2007). According to Qi et al. (2007), β-amyloid peptides, 1–40 and 1–42 were found in post-ischemic hippocampus. Hippocampal and cortical neuronal cells were the most intensely stained cells in the post-ischemic human brains. Additionally, evidence from clinical investigations showed that plasma amyloid was raised in cases with ischemic brain injury (Lee et al., 2005; Zetterberg et al., 2011; Liu et al., 2015). The increase of amyloid in serum correlated negatively with the clinical outcome following an ischemic brain episode (Zetterberg et al., 2011).

Following focal brain ischemia, messenger RNA (mRNA) of the amyloid protein precursor increased from 150 to 200% after 7 days of survival (Shi et al., 1998, 2000). The amyloid protein precursor is cleaved *via* α-secretase along a non-amyloidogenic pathway. Following experimental ischemic brain episode, the α-secretase mRNA was reduced (Nalivaeva et al., 2004; Yan et al., 2007; Pluta et al., 2020a). In the amyloidogenic pathway, the amyloid protein precursor is cleaved *via* β- and γ-secretase to form amyloid (Wen et al., 2004a; Tabaton and Tamagno, 2007; Pluta et al., 2013a; Guo et al., 2021). Available data suggest that ischemia activates mRNA of β-secretase (Blasko et al., 2004; Chen et al., 2004; Wen et al., 2004a; Chuang et al., 2008; Ye et al., 2009). Presenilins mRNA, which are induced *via* brain ischemia (Tanimukai et al., 1998; Pennypacker et al., 1999), are involved in the generation of amyloid by the γ-secretase complex (Polavarapu et al., 2008). The above evidence helps us understand the progressive neuronal death after an episode of brain ischemia, accumulation of amyloid, and the slow progressive development of Alzheimer's disease type neurodegeneration (Pluta et al., 2009, 2010, 2020b,c,d). In studies on post-ischemic brain changes, elevated presenilin 1 mRNA in the CA3 area and dentate gyrus of hippocampal neurons was noted at 3 days (Tanimukai et al., 1998). In another study, increased presenilin mRNAs were noted in the hippocampus, striatum, and the brain cortex following focal ischemia (Pennypacker et al., 1999). Presenilin mRNAs showed a maximum increase in the hippocampus and cortex. The cortex exhibited an increase of presenilin 1 and 2 mRNAs within 1–8 days after local ischemia with recirculation (Pennypacker et al., 1999), whereas, the hippocampus exhibited upregulation of presenilin 1 and 2 mRNAs within 4–8 days after ischemia (Pennypacker et al., 1999). The elevated level was greater on the contralateral side than on the side of focal ischemic brain injury. This difference is most likely associated with the loss of brain neurons expressing presenilin mRNAs on the ischemic side (Pennypacker et al., 1999).

In the CA1 subfield of the hippocampus, 2 days after ischemia, the expression of the amyloid protein precursor gene was below the control value (Table 1) (Kocki et al., 2015). Within 7–30 days after ischemia with reperfusion, the expression of the amyloid protein precursor gene was above the control value (Table 1) (Kocki et al., 2015). The expression of the β-secretase gene was above the control value, 2–7 days after ischemia in the CA1 region (Table 1) (Kocki et al., 2015). Thirty days after ischemia, the expression of β-secretase gene was below the control value (Table 1) (Kocki et al., 2015). In the CA1 area of the hippocampus, the expression of presenilin 1 and 2 genes increased within 2–7 days after ischemia (Table 1) (Kocki et al., 2015). In contrast, 30 days following ischemia, the expression of presenilin 1 and 2 genes was below the control value (Table 1) (Kocki et al., 2015).

**Table 1:** Changes in the expression of the Alzheimer's disease-linked genes in the CA1 region of the hippocampus at various times after experimental ischemic brain injury.

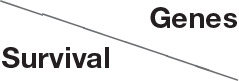	**APP**	**BACE1**	**PSEN1**	**PSEN2**	**MAPT**
2 days	↓	↑	↑	↑	↑
7 days	↑	↑	↑	↑	↓
30 days	↑	↓	↓	↓	↓

*Expression: ↑, increase; ↓, decrease. Genes: APP, amyloid protein precursor; BACE1, β-secretase; PSEN1, presenilin 1; PSEN2, presenilin 2; MAPT, Tau protein*.

At 2, 7, and 30 days following ischemia, the expression of the amyloid protein precursor gene was found to be above the control value in the CA3 region (Table 2) (Pluta et al., 2020a). In this area, the α-secretase gene expression was below the control value at 2, 7, and 30 days after ischemia (Table 2) (Pluta et al., 2020a). The expression of the β-secretase gene was below the control value following ischemia in the CA3 area, in 2–7 days (Table 2). In contrast, 30 days after ischemia, the β-secretase gene expression was above the control value (Table 2) (Pluta et al., 2020a). In the CA3 subfield, the expression of the presenilin 1 gene increased in 2–7 days following ischemia (Table 2). In 30 days after ischemia, the expression of the presenilin 1 gene was below the control value (Table 2) (Pluta et al., 2020a). In contrast, the expression of the presenilin 2 gene was reduced 2–7 days after ischemia (Table 2). But 30 days after ischemia, the expression of the presenilin 2 gene was above the control value (Table 2) (Pluta et al., 2020a).

**Table 2:** Changes in the expression of the Alzheimer's disease-linked genes in the CA3 region of the hippocampus at various times after experimental ischemic brain injury.

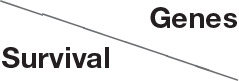	**APP**	**ADAM10**	**BACE1**	**PSEN1**	**PSEN2**	**MAPT**
2 days	↑	↓	↓	↑	↓	↓
7 days	↑	↓	↓	↑	↓	↑
30 days	↑	↓	↑	↓	↑	↑

*Expression: ↑, increase; ↓, decrease. Genes: APP, amyloid protein precursor; ADAM10, α-secretase; BACE1, β-secretase; PSEN1, presenilin 1; PSEN2, presenilin 2; MAPT, Tau protein*.

In the medial temporal cortex, the expression of the amyloid protein precursor gene was below the control value, 2 days after ischemia (Table 3) (Pluta et al., 2016a). In the above area, in 7–30 days after ischemia, the expression of the amyloid protein precursor gene was above the control value (Table 3) (Pluta et al., 2016a). The β-secretase gene expression was above the control value at 2 days after ischemia (Table 3) (Pluta et al., 2016a). But in 7–30 days after ischemia, the expression of the β-secretase gene was reduced (Table 3) (Pluta et al., 2016a). The expression of the presenilin 1 gene was lowered below the control value, while the presenilin 2 gene was above the control value 2 days after ischemia (Table 3) (Pluta et al., 2016b). Seven days after ischemia, the expression of the presenilin 1 gene was reduced, while that of the presenilin 2 gene was increased (Table 3) (Pluta et al., 2016b). Thirty days after ischemia, the expression of the presenilin 1 gene was above the control value, while that of the presenilin 2 gene was below the control value (Table 3) (Pluta et al., 2016b).

**Table 3:** Changes in the expression of the Alzheimer's disease-linked genes in the medial temporal cortex at various times after experimental ischemic brain injury.

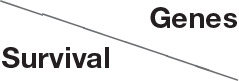	**APP**	**BACE1**	**PSEN1**	**PSEN2**
2 days	↓	↑	↓	↑
7 days	↑	↓	↓	↑
30 days	↑	↓	↑	↓

*Expression: ↑, increase; ↓, decrease. Genes: APP, amyloid protein precursor; BACE1, β-secretase; PSEN1, presenilin 1; PSEN2, presenilin 2*.

Data show that brain ischemia triggers neuronal death in the hippocampus and in the medial temporal cortex in conjunction with amyloid, defining a new process that regulates the survival of neurons and/or death after-ischemia (Pluta et al., 1994b, 1997a, 2009, 2016a,b; Hall et al., 1995; Jendroska et al., 1995, 1997; Palacios et al., 1995; Ishimaru et al., 1996; Wisniewski and Maslinska, 1996; Yokota et al., 1996; Qi et al., 2007; Kocki et al., 2015; Pluta et al., 2020a; Pluta and Ułamek-Kozioł, 2021).

## Tau Protein in Post-Ischemic Neurodegeneration

Strong neuronal staining of tau protein was found following ischemia in the hippocampus and the brain cortex (Dewar et al., 1993, 1994; Geddes et al., 1994; Sinigaglia-Coimbra et al., 2002). Also, tau protein staining was documented in post-ischemic microglia, astrocytes, and oligodendrocytes (Dewar and Dawson, 1995; Irving et al., 1997; Uchihara et al., 2004; Majd et al., 2016; Fujii et al., 2017). Evidence shows that after ischemia, the hyperphosphorylated tau protein dominates in neuronal cells and goes along with apoptosis (Wen et al., 2004b,c; Wen et al., 2007; Majd et al., 2016; Fujii et al., 2017; Basurto-Islas et al., 2018). The above data point out that neuronal apoptosis following ischemia is connected with hyperphosphorylation of tau protein. In addition, it is also evident that ischemic brain injury was engaged in paired helical filaments (Khan et al., 2018), neurofibrillary tangle-like (Wen et al., 2004b,c; Wen et al., 2007), and neurofibrillary tangles development (Kato et al., 1988; Hatsuta et al., 2019). Tau protein was detected in the plasma samples of humans after ischemic brain injury and it most probably indicated the progress of neuronal changes after ischemia (Bitsch et al., 2002; Kurzepa et al., 2010; Bielewicz et al., 2011; Mörtberg et al., 2011; Randall et al., 2013; Lasek-Bal et al., 2016; De Vos et al., 2017).

## Dysregulation of Tau Protein Gene in Post-Ischemic Neurodegeneration

In the CA1 area of the hippocampus, the expression of the tau protein gene was found to be increased above the control value, 2 days after ischemia (Table 1) (Pluta et al., 2018). In contrast, at 7–30 days after brain ischemia, gene expression was found to be below the control value (Table 1) (Pluta et al., 2018). In the CA3 subfield, the expression of the tau protein gene was found to be below the control value, 2 days after ischemia (Table 2) (Pluta et al., 2020a). But, within 7–30 days following ischemia, the expression of the tau protein gene was higher than the control value (Table 2) (Pluta et al., 2020a). The data indicate that post-ischemic brain injury triggers neuronal damage and death in the hippocampus in a tau protein-dependent mechanism, defining a new process, which in the long run regulates neuronal survival and/or death following ischemia (Geddes et al., 1994; Dewar and Dawson, 1995; Wen et al., 2004b, 2007; Majd et al., 2016; Fujii et al., 2017; Basurto-Islas et al., 2018; Khan et al., 2018; Pluta et al., 2018, 2020a; Pluta and Ułamek-Kozioł, 2021).

## Dementia in Post-Ischemic Neurodegeneration

Many studies have documented the development of dementia in animals after ischemic brain injury with recirculation (De la Tremblaye and Plamondon, 2011; Kiryk et al., 2011; Li et al., 2011; Pluta et al., 2011; Cohan et al., 2015). Locomotor hyperactivity was noted after experimental brain ischemia (Kuroiwa et al., 1991; Karasawa et al., 1994) as in Alzheimer's disease. A lengthening of ischemic episode causes a longer duration of motor hyperactivity, and this is positively correlated with an increased number of damaged and lost neurons, especially in the hippocampus and progressive inflammation in the brain (Pluta et al., 2010; Kiryk et al., 2011; Sekeljic et al., 2012; Radenovic et al., 2020). Additionally, post-ischemic brain damage causes loss of reference and working memory with the progress of a spatial memory deficit (Kiryk et al., 2011). The progress of cognitive deficit develops systematically along with the lengthening of post-ischemic time (Kiryk et al., 2011). After repeated experimental brain ischemia with recirculation, durable motor hyperactivity with cognitive deficits and reduced anxiety was presented (Ishibashi et al., 2006). The development of dementia was related to general brain atrophy (Hossmann et al., 1987; Pluta, 2000a, 2002a,b; Pluta et al., 2009, 2012b,c; Jabłoński et al., 2011). In these cases learning and memory deficits in experimental post-ischemic brain neurodegeneration irreversibly progressed and persisted forever (Kiryk et al., 2011).

The progressive development of dementia is a patients dangerous consequence of post-ischemic pathology (Gemmell et al., 2012, 2014; Brainin et al., 2015; Mok et al., 2016; Portegies et al., 2016; Kim and Lee, 2018). The occurrence of dementia after the first ischemic stroke and recurrent stroke is calculated roughly at 10 and 41%, respectively (Pendlebury and Rothwell, 2009). Within a 25-year follow-up, the incidence of dementia has been calculated approximately at 48% (Kokmen et al., 1996). Worldwide, dementia after ischemic stroke occurs between 5 and 50% of cases, depending on diagnostic criteria, population demographics, and geographical location (Surawan et al., 2017). In fact, it is certain that dementia in post-ischemia brain neurodegeneration has many risk factors in common with the development of dementia in sporadic cases of Alzheimer's disease (Thal et al., 2003). It is highly likely that post-ischemic brain neurodegeneration may precede the final development of Alzheimer's disease.

## Conclusion

**Figure 1:**
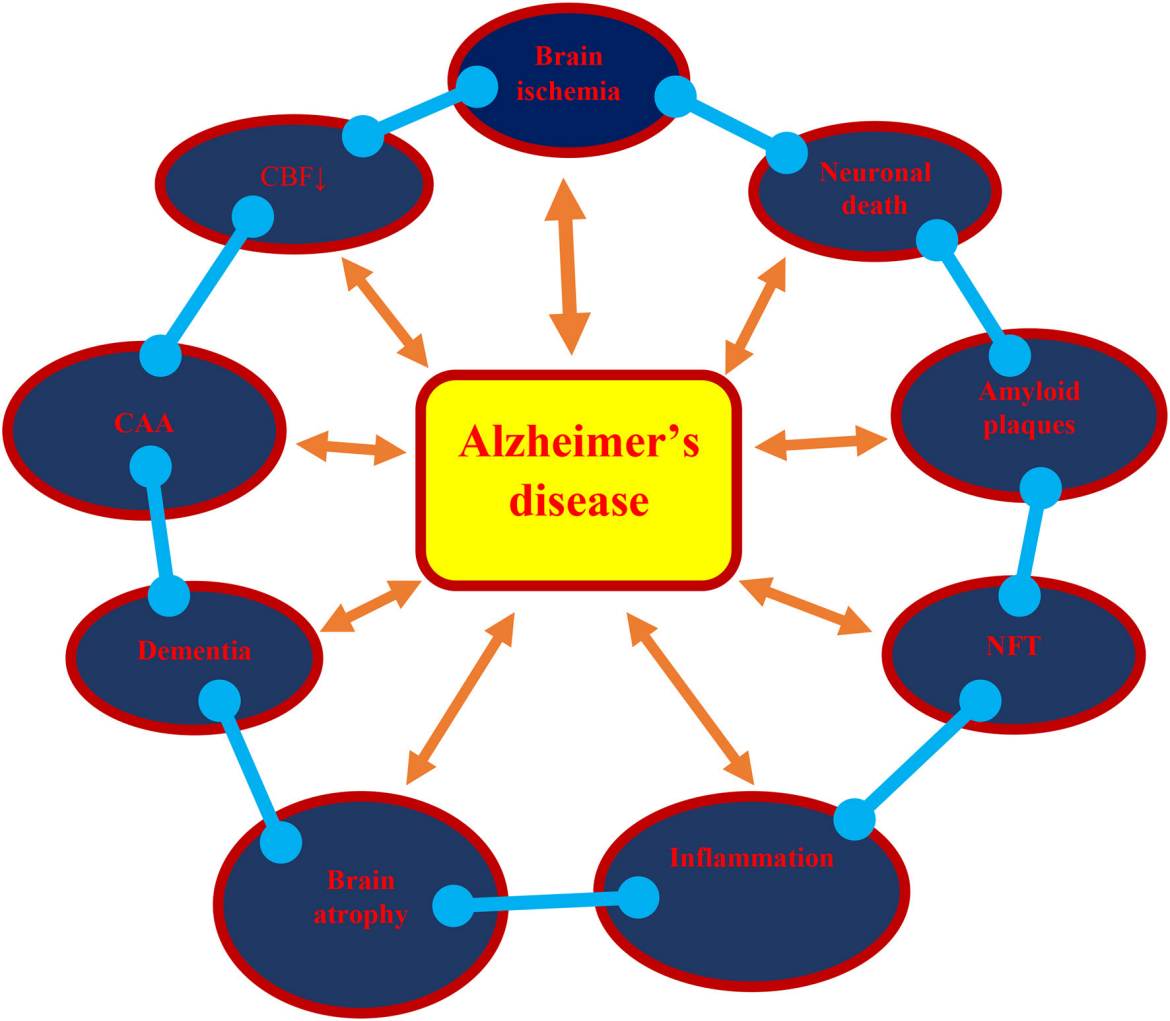
A possible vicious circle in the development of Alzheimer's disease as a result of brain ischemia. **↓**, decrease; CAA, cerebral amyloid angiopathy; CBF, cerebral blood flow; NFT, neurofibrillary tangles.

This review presents the phenotype and genotype of Alzheimer's disease in post-ischemic brain neurodegeneration, such as neuropathology, amyloid, dysfunctional tau protein, and their genes, which altogether play an important role in the development of full-blown dementia (Figure 1). It provides Alzheimer's disease-linked gene changes of the amyloid protein precursor, α-secretase, β-secretase, presenilin 1, presenilin 2, and tau protein in experimental post-ischemic injury in the CA1 and CA3 areas of the hippocampus and medial temporal cortex. The evidence demonstrates that post-ischemic brain damage causes the death of neuronal cells in the hippocampus and temporal cortex in a manner that is dependent on the amyloid and dysfunctional tau protein. The above changes are associated with the accumulation of the amyloid in brain cells and in the extracellular spaces, such as diffuse and senile amyloid plaques, and the massive loss of neuronal cells with general brain atrophy, especially in the hippocampus, which finally leads to full-blown dementia in Alzheimer's disease (Figure 1). It is likely that following ischemia, the development of amyloid plaques in the brain is caused by an increased amyloid generation, an intensified inflow from blood, and worsening of amyloid clearance from the brain tissue. Based on the presented evidence, it can be concluded that post-ischemic brain neurodegeneration influences the processing of the amyloid protein precursor, at both the gene and protein level, and leads to the generation of amyloid plaques in brain parenchyma.

In addition, observations showed that ischemic injury of the CA1 and CA3 areas of the hippocampus influence the expression of the tau protein gene. It can also be noted that dysregulated tau protein takes part in neuronal cell death in the CA1 and CA3 areas of the hippocampus. The existing data documented the development of neurofibrillary tangles following ischemia (Figure 1). In support of the above fact, elevated levels of cyclin-dependent kinase 5, which are involved in the development of neurofibrillary tangle-like tauopathy, were found after experimental ischemic brain injury (Wen et al., 2007). This may suggest the linking of dysfunctional tau protein with the onset of neuronal cell death in the hippocampus after ischemia. The above evidence also indicates the regulation of the ischemic death of neuronal cells in the CA1 and CA3 areas of the hippocampus in a manner dependent on the structure of the tau protein.

The parallelism between post-ischemic brain neurodegeneration and Alzheimer's disease at the molecular level (Figure 1) is remarkable. The conclusions drawn from this investigation of ischemia-triggered Alzheimer's disease-like neuropathology, proteins, and their genes indicates that they contribute to the death of neuronal cells, the generation of the amyloid plaques, the development of neurofibrillary tangles, and finally, neurodegeneration with full-blown dementia (Figure 1). These findings may significantly contribute to finding new causal treatments of post-ischemic brain neurodegeneration and Alzheimer's disease.

The dominant hypothesis of the etiology of Alzheimer's disease, neuropathological guidelines for the diagnosis of the disease, and most of the wide-ranging therapeutic efforts, both in research and clinical practice, have been built around the amyloid and tau proteins as causal factors (Ittner and Ittner, 2018; Busche and Hyman, 2020; Uddin et al., 2020). However, the causal link between amyloid and the development of Alzheimer's disease remains unproven. Currently, in the context of a comprehensive evaluation of the past and contemporary research, critical questions are being raised regarding the role of amyloid and tau protein in the diagnosis, etiology, and definition of Alzheimer's disease. It is argued that a holistic review of the available data does not allow a clear conclusion for the fact that amyloid plays a central or unique role in the development of Alzheimer's disease. A new analysis of data proposes that deposits of amyloid and dysfunctional tau protein may not be the primary cause in the pathogenesis of Alzheimer's disease; further research is needed in this field (Tse and Herrup, 2017; Jack et al., 2018; Morris et al., 2018). Regarding the two potentially dangerous substances blamed for the green light in Alzheimer's disease, recent data show that amyloid and tau protein pathology should be triggered by some factors and then interact with each other, exerting a synergistic deleterious effect on the neural network, which is believed to initiate the progression of Alzheimer's disease (Morris et al., 2018). It is certain that alterations of amyloid and tau protein are currently ruled out as the sole cause of dementia, as it does not explain why about half of the world's population over the age of 45 have amyloid plaques and neurofibrillary tangles without dementia (Katzman, 1988; Price et al., 1992; Rowe et al., 2007; Jagust et al., 2009; Knopman et al., 2013; Atlante et al., 2020). The presence of amyloid plaques, which do not cause any disturbance, has been found in older adults who identify themselves with excellent cognitive functions (Katzman, 1988; Price et al., 1992; Rowe et al., 2007; Jagust et al., 2009; Knopman et al., 2013; Atlante et al., 2020). In these subjects, the average neurofibrillary tangle concentration was observed to increase exponentially with age (Price and Morris, 1999). To sum up, there are no older adults who do not exhibit the presence of amyloid plaques and neurofibrillary tangles in their brains (Garrett, 2018). Additionally, the appearance of the hippocampal atrophy in cognitively normal elderly may not be dependent on amyloidosis (Chételat, 2013). On the other hand, a multicenter study reported that patients diagnosed with Alzheimer's disease were found to be negative for brain amyloid; for example, amyloid was noted to be absent in 32% of cases studied (Doraiswamy et al., 2012).

After analyzing past and contemporary studies about on the amyloid theory of Alzheimer's disease by Morris et al. (2018), the following four important conclusions were drawn: (1) Amyloid plaques and neurofibrillary tangles may be present in the brain without cognitive impairment. (2) The clinical diagnosis of Alzheimer's disease does not include the structure of amyloid and tau protein in the brain. (3) The number, size, and enlargement of amyloid plaques are not related to cognitive impairment. (4) Soluble amyloid and amyloid plaques in the brain are not a warning sign of impending dementia. Novel discoveries have demonstrated that amyloid and neurofibrillary tangles do not initiate Alzheimer's disease as these are only two among a multitude of degenerative changes that occur in this disease (Gauthier et al., 2018; Kalvach and Vogner, 2019). Our review aims to challenge these shared views and suggests that continuing this approach may be counterproductive. Instead, the data suggest that we should look for alternative views on the etiology of Alzheimer's disease that are currently potentially under consideration. We propose that a thorough understanding of the etiology of Alzheimer's disease and the implementation of the final, effective treatment requires a new objective approach in this topic, beyond the current amyloid-centric approach, without excluding the role of amyloid. The continued interest in the research on cerebral ischemia provides evidence that ischemia may be involved in the development of the genotype and phenotype of Alzheimer's disease, suggesting that cerebral ischemia can be considered a useful model for understanding the mechanisms underlying the development of Alzheimer's disease. In spite of reasonable doubts about the role of brain ischemia in the etiology of Alzheimer's disease, the mounting evidence on the ischemic development of the disease should not be ignored. Ignoring the huge number of experimental and clinical evidence on the connection between ischemic brain injury and Alzheimer's disease will not only hamper proper understanding of the disease pathways, but also development strategies for diagnosis, management, and therapy of Alzheimer's disease. Although significant progress has been made in the last few years, it is clear from a review of the available publications that much remains to be clarified regarding the relationship between cerebral ischemia and Alzheimer's disease. The fact that even the combined association of amyloid and tau protein pathology does not necessarily lead to Alzheimer's disease, in addition to the fact that other factors like ischemia might be playing a role in triggering or accelerating the Alzheimer's disease type cognitive decline, has prompted a handful of investigators to conclude that studies should aim to understand the interactions among these factors during the progression of injury following brain ischemia, and that the genetic, molecular, and cognitive profiles of patients must be analyzed on an individual basis (Gauthier et al., 2018). In conclusion, the behavior of Alzheimer's disease-related genes in the human brain after ischemia should be investigated in the near future as the lack of such data is a significant limitation in our presentation.

## Author Contributions

RP: idea of MS, preparing, and editing. SJ: searching for literature and preparing figure and tables. SC: preparing and editing MS. All authors: contributed to the article and approved the submitted version.

## Conflict of Interest

The authors declare that the research was conducted in the absence of any commercial or financial relationships that could be construed as a potential conflict of interest.
